# B-Type Natriuretic Peptide as a Predictor of Short-Term Mortality in On-Pump Coronary Artery Bypass Grafting

**DOI:** 10.21470/1678-9741-2017-0154

**Published:** 2017

**Authors:** Jamil Alli Murad Junior, Maurício Nassau Machado, Marcio Pimentel Fernandes, Marcelo José Ferreira Soares, Ingrid Helen Grigolo, Cristiane Carvalho Singulane, Moacir Fernandes de Godoy

**Affiliations:** 1 Faculdade de Medicina de São José do Rio Preto (FAMERP), São José do Rio Preto, SP, Brazil

**Keywords:** Natriuretic Peptides, Coronary Artery Bypass, Prospective Studies

## Abstract

**Objective:**

The present study refers to a determination of the preoperative B-type natriuretic peptide is a predictor of short-term all-cause mortality in patients undergoing on-pump coronary artery bypass graft surgeries.

**Methods:**

Two hundred and twenty-one patients undergoing on-pump coronary artery bypass graft surgeries were evaluated prospectively during a 30-day postoperative follow-up period. Serum B-type natriuretic peptide concentration was measured without a 24-hour period prior to the surgical procedure and the value obtained was correlated with a short-term all-cause mortality.

**Results:**

Data analysis showed that all-cause mortality rates were equal to 9.5% in 30 days. Accuracy analysis by the receiver operating characteristic curve found an ideal cut-off value of B-type natriuretic peptide equal to 150 pg/mL in relation to mortality (AUC=0.82, 95% CI=0.71-0.87, *P*<0.001). Multivariate analysis showed that B-type natriuretic peptide value greater than or equal to 150 pg/mL (*P*=0.030, HR=3.99, 95% CI=1.14-13.98) was an independent predictor of all-cause mortality in a 30-day follow-up period.

**Conclusion:**

Preoperative serum B-type natriuretic peptide concentration is an independent predictor of short-term all-cause mortality in patients undergoing coronary artery bypass grafting with cardiopulmonary bypass.

**Table:** 

Abbreviations, acronyms & symbols	
BNP	= B-type natriuretic peptide
CABG	= Coronary artery bypass grafting
HR	= Hazard ratio
OPCABG	= On-pump coronary artery bypass grafting
ROC	= Receiver operating characteristic

## INTRODUCTION

Since its introduction in the 1960s, coronary artery bypass grafting (CABG) is the most studied surgical procedure in medical history and rapidly as the standard treatment for patients with extensive coronary artery disease^[1]^.

In the last decades, there has been a significant decline in short-term mortality for this procedure. This work can be attributed to an improvement in the surgical technique combined with the significant progress in perioperative care, as well as the development and diffusion of databases that provide a better definition and understanding of variables that impact on mortality after CABG^[2-6]^.

This stabilization of mortality is a challenge for the discovery of new variables to predict mortality in order to further improve the care related to this procedure. In this context, due to its close correlation with ventricular parietal stress and clinical and hemodynamic variables that determine a higher cardiovascular risk, B-type natriuretic peptide (BNP) has been investigated as a new prognostic tool to predict mortality in cardiac surgery^[7-9]^.

In this study, the objective is to evaluate whether the preoperative BNP concentration is an independent predictor of short-term all-cause mortality in patients undergoing on-pump coronary artery bypass grafting (OPCABG).

## METHODS

Patients consecutively submitted to OPCABG admitted to the Intensive Care Unit in the postoperative period of cardiac surgeries at Hospital de Base of São José do Rio Preto (SP, Brazil). Patients younger than 18 years of age, advanced neoplasia or those who underwent a combined cardiac procedure, congenital surgery, cardiac transplantation, aortic surgery, valve surgery and off-pump coronary artery bypass surgery were excluded.

The OPCABG were performed by median transsternal thoracotomy with extracorporeal circulation, aorta-bicaval cannulation, transverse aortic clamping and antegrade and retrograde cardioplegia using normothermic blood solution. Patients were treated according to European and American guidelines in force at the time^[10]^.

The data were prospectively collected through a computerized database of the Hospital de Base of São José do Rio Preto for an average follow-up period of 30 days. The study protocol was approved by the local ethics committee (CAAE 53306916.8.0000.5415).

Variables included in the study were demographic, preoperative comorbidities, degree of left ventricular systolic dysfunction, intraoperative variables, and all-cause mortality in 30 days. The definition of all variables, as well as the indication and performance of the surgical procedures are based on the guidelines published by the European Society of Cardiology and European Association for Cardio-Thoracic Surgery^[10]^.

The serum concentration of BNP was measured in the immediate preoperative period (24 hours before surgery) in all surgeries by the electrochemiluminescence method using the Siemens ADVIA Centaur equipment (Siemens Medical).

### Statistical Analysis

Categorical variables were presented in absolute numbers (percentages) and the continuous variables in mean and standard deviation or median and interquartile ranges (Q3-Q1), according to the type of distribution.

Statistical analysis was performed with SPSS software (version 20.0). The power to discriminate the preoperative serum concentration of BNP to predict all-cause mortality within 30 days after surgery was determined by calculating the area under the Receiver Operating Characteristic (ROC) curve, value > 0.60 considered as not determined by chance.

In this study, the risk analysis with the Cox proportional regression model was used to establish the relationship between the dependent variable (all-cause mortality in 30 days) and the exploratory variables. Univariate variables related to mortality (*P*≤0.05) were considered for multivariate regression analysis. Independent predictors of mortality were established for the variables with *P*≤0.05 in the multivariate analysis.

The probability of survival during the 30-day follow-up period was estimated by the Kaplan-Meier method. The log rank test was used to compare the probability of survival.

Values with *P*≤0.05 were considered statistically significant.

## RESULTS

**Fig. 1:**
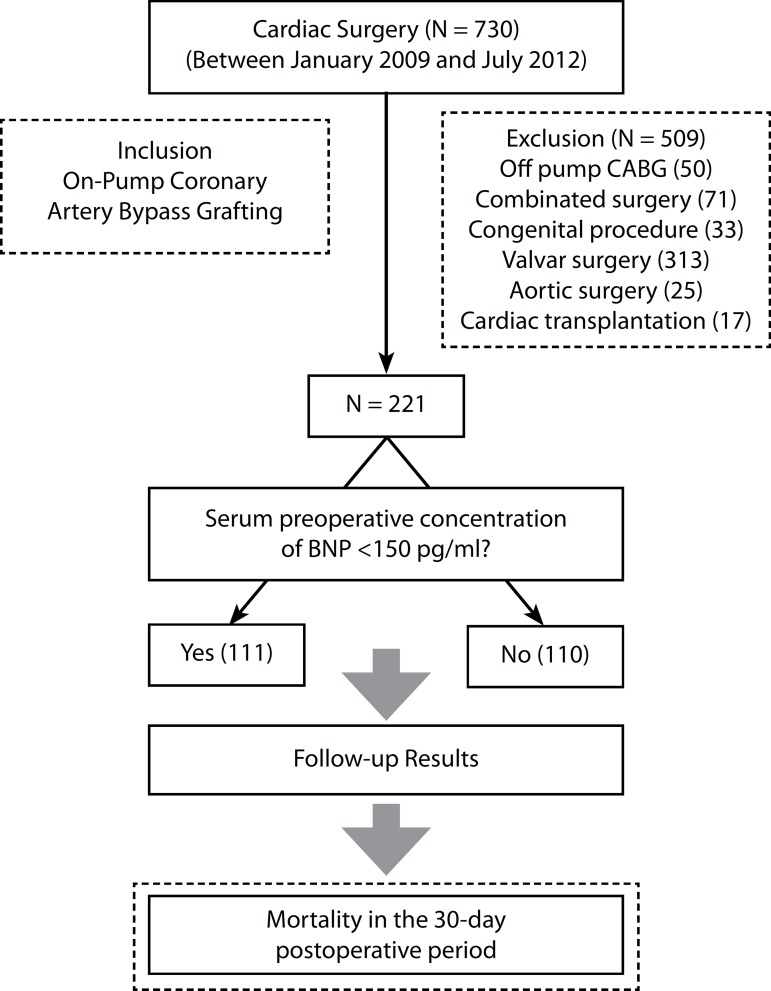
Flowchart for study enrollment and patient outcome (CABG - Coronary Artery Bypass Graft Surgery, BNP - B -type natriuretic peptide, PO - postoperative).

In the period evaluated, a total of 730 patients were consecutively submitted to open cardiac surgical procedures. Two hundred twenty-one patients underwent OPCABG were included in the study with a 30-day follow-up period in all patients. A total of 509 patients who underwent combined cardiac procedures (71 patients), congenital surgery (33 patients), cardiac transplantation (17 patients), aortic surgery (25 patients), valve surgery (313 patients) and off pump CABG (50 patients) (Figure 1).

The baseline characteristics of patients undergoing OPCABG evaluated in this study are summarized in Table 1. The median age was 60 (52-67) years and 66% were men. Among the 221 patients, 70 (32%) were diabetic, 192 (87%) had systemic arterial hypertension. Eighty-two (37%) patients had urgency or emergency surgery, 74 (33%) patients were operated in the presence of acute coronary syndrome, 43 (19%) patients had moderate or significant left ventricular dysfunction. Intra-aortic balloon pump was implanted in the preoperative period in 13% of the patients and the mean duration of cardiopulmonary bypass was 94 minutes.

**Table 1:** Baseline characteristics of patients undergoing myocardial revascularization surgery (N=221), overall and stratified according to the cutoff value of BNP (150 pg/ml).

Baseline characteristics	All patients (221)	BNP < 150 pg/mL (111)	BNP ≥ 150 pg/mL (110)	*P* Value
	Median (25^th^ - 75^th^) or N (%)			
Age (years)	60 (52 - 67)	58 (51 - 66)	62 (56 - 69)	0.014
Male gender	146 (66)	75 (68)	71 (65)	0.635
Weight (kg)	72 (65 - 83)	75 (67 - 85)	70 (63 - 80)	0.023
Height (m)	1,66 (1.60 - 1.70)	1.66 (1.60 - 1.70)	1.65 (1.60 - 1.70)	0.772
Body mass index (kg/m^2^)	27 (24 - 30)	28 (25 - 31)	26 (24 - 29)	0.010
ICU Length of Stay	3 (2 - 5)	3 (2 - 5)	3 (2 - 6)	0.342
Acute Coronary Syndrome	74 (33)	22 (20)	52 (47)	<0.001
Unstable Angina	37 (50)	16 (73)	21 (40)	<0.001
Non-ST Elevation MI	22 (30)	4 (18)	18 (35)	
ST Elevation MI	15 (20)	2 (9,0)	13 (25)	
LMCA > 50%	59 (27)	29 (26)	30 (27)	0.912
Number of grafts	3 (2 - 3)	3 (2 - 3)	3 (2 - 3)	0.197
Urgency/Emergency	82 (37)	23 (21)	59 (54)	<0.001
COPD	7 (3.2)	3 (2,7)	4 (3,6)	0.722
High blood pressure	192 (87)	93 (84)	99 (90)	0.171
Diabetes mellitus	70 (32)	26 (23)	44 (40)	0.008
Baseline SCr (mg/dL)	1,10 (0,90 – 1.30)	1,10 (1,00 – 1.30)	1.10 (0.90 – 1.40)	0.761
LV dysfunction	43 (19)	11 (9,9)	32 (29)	<0.001
CPB time (min)	94 (77 - 111)	94 (78 - 110)	93 (77 - 113)	0.763
IABP	29 (13)	9 (8,1)	20 (18)	0.027
Additive EuroSCORE	2 (1 - 4)	2 (0 - 3)	3 (1 - 5)	<0.001
Low (0 - 2)	115 (52)	72 (65)	43 (39)	<0.001
Intermediate (3 - 5)	73 (33)	33 (30)	40 (36)	
High (6 - 8)	21 (9.5)	4 (3.6)	17 (15)	
Very high (>9)	12 (5.4)	2 (1.8)	10 (9.0)	

BNP = B-type natriuretic peptide; COPD=chronic obstructive pulmonary disease; CPB=cardiopulmonary bypass; IABP=intra-aortic balloon pump; ICU=intensive care unit; LMCA=left main coronary artery; LV=left ventricle; MI=myocardial infarction; SCr=Serum creatinine

### Mortality from all Causes in the 30-day Follow-up Period

**Fig. 2:**
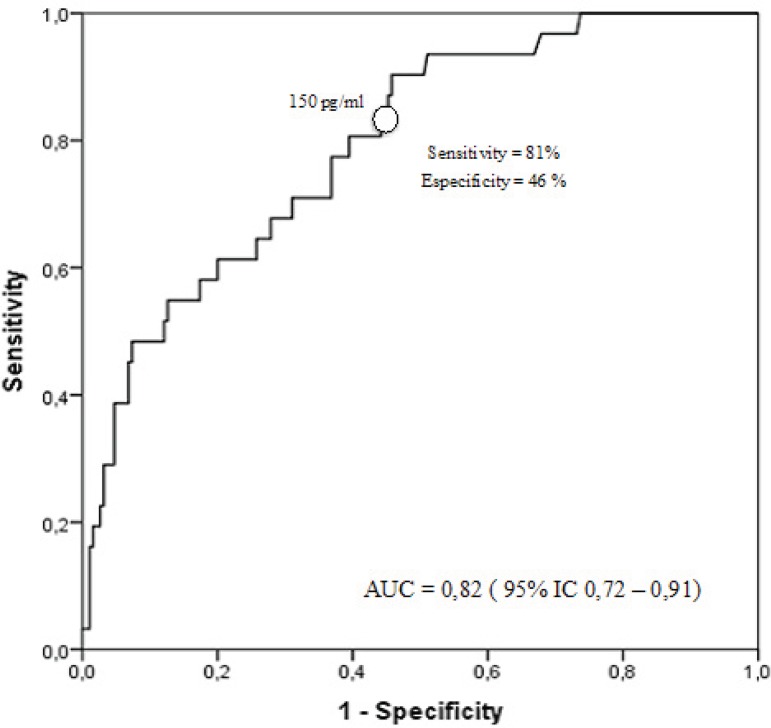
ROC curve showing point of maximum sensitivity and specificity correlating BNP measured in the preoperative and all-cause mortality during the 30-day postoperative period.

There were 21 (9.5%) deaths in 30 days. The optimum cutoff value selected by ROC curve was 150 pg/mL, the area under the curve was equal to 0.82 (CI 95% 0.72 to 0.91), with a sensitivity of 81% and a specificity of 46%, positive and negative predictive values, respectively, equal to 24% and 95% for all-cause mortality in 30 days after CABG (Figure 2).

When stratified according to the optimal cutoff value of the preoperative serum BNP concentration equal to 150 pg/dl, the group of patients with BNP greater than or equal to 150 mg/dl was older (58 years *versus* 62 years, *P*=0.010), higher body mass index (28 kg/m^2^ *versus* 26 kg/m^2^, *P*=0.010), in the presence of acute coronary syndrome (20% *versus* 47%, *P*<0.0010, urgency/emergency surgery 21% *versus* 54%, *P*<0.001), higher rate of the left ventricular dysfunction (9.9% *versus* 29%, *P*<0.001), intra-aortic balloon use (8.1% *versus* 18%, *P*=0.027) and higher EuroSCORE additive (2 *versus* 3, *P*<0.001) (Table 1).

Table 2 shows the variables univariate associated with mortality in 30 days, as well as the independent predictors determined by the multivariate Cox regression analysis. In order of prognostic importance, the time of extracorporeal circulation (*P*=0.027, HR=1.02, 95% CI 1.00- 1.03), age (*P*<0.001, HR=1.1, 95% CI 1.04-1.16), preoperative serum creatinine (*P*<0.001, HR = 1.95, 95% CI 1.42-2.68), BNP greater than or equal to 150 pg/ml (*P*=0.030, HR=3.99, 95% CI 1.14-13.98) were independent predictors of all-cause death in the 30-day postoperative followup period.

**Table 2:** Univariate and multivariate analysis by the Cox Regression model for mortality in the 30-day postoperative period.

All patients	Univariate			Multivariate		
	HR	95%CI	*P* Value	HR	95%CI	*P* Value
Age (years)	1.10	1.05 – 1.16	<0.001	1.10	1.04 – 1.16	<0.001
Gender (male)	1.04	0.42 – 2.58	0.930			
Urgency/emergency surgery	5.94	2.17 – 16.21	0.001			
Baseline SCr (mg/dL)	2.44	1.80 – 3.30	<0.001	1.95	1.42 – 2.68	<0.001
LV Dysfunction	2.77	1.15 – 6.70	0.023			
Cardiopulmonary bypass time (min)	1.03	1.01 – 1.04	<0.001	1.02	1.00 – 1.03	0.027
BNP ≥ 150 pg/mL	6.55	1.93 – 22.23	0.003	3.99	1.14 – 13.98	0.030

BNP=B-type natriuretic peptide; LV=left ventricle; SCr=Serum creatinine

**Fig. 3:**
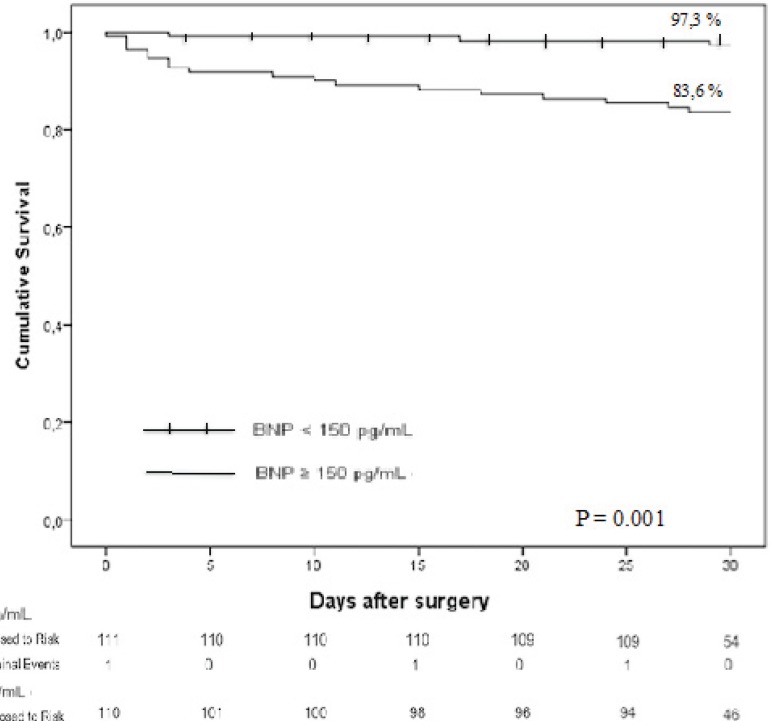
Kaplan Meier survival curves for patients undergoing CABG stratified for serum preoperative BNP.

The survival probability of patients undergoing OPCABG was 90.5% in the 30-day postoperative follow-up period. Survival for patients with BNP ≥ 150 pg/ml in 30 days was 83.6% and for those with BNP <150 pg/ml was 97.3% (*P*<0.001, Figure 3).

## DISCUSSION

This prospective study with a significant cohort revealed independent predictors of all-cause mortality in patients undergoing OPCABG. In ascending order of prognostic importance, the factors were time of extracorporeal circulation, age, preoperative serum creatinine, and preoperative serum BNP concentration ≥ 150 pg/ml.

Predictors of short-term mortality in CABG have been well established in the last decades. Consequently, risk stratification systems were created with the objective of predicting shortterm mortality after CABG and the most employed in the USA and Europe are, respectively, the STS risk score and EuroSCORE II. Widely validated in thousands of patients in a multicenter database, these systems have contributed to a progressive decline in mortality in the short-term and are recommended as a gold standard model for risk stratification in CABG^[8,11-15]^. It is necessary to identify new variables capable of improving and better predict short-term results in myocardial revascularization surgery. In this context, the role of preoperative serum BNP concentration as a risk stratification instrument in cardiac surgery has been investigated and further studies are needed to determine the actual clinical usefulness of BNP in relation to already validated risk stratification systems^[16,17]^.

In our study, preoperative serum BNP concentration greater than 150 pg/mL was an independent predictor of mortality with the hazard ratio (HR) function equal to 3.96 (*P*=0.006, 95% CI 1.50 - 10.50) and a significant reduction in the probability of survival in 30 days. In patients undergoing OPCABG, the use of preoperative serum BNP concentration had already been evaluated as a predictor of adverse clinical events. Bernstein et al.^[18]^ previously demonstrated that the preoperative serum concentration of high BNP was associated with a higher incidence of postoperative atrial fibrillation and longer hospital stay. In a study by Cuthbertson et al.^[19]^, a higher preoperative concentration of BNP was a predictor of greater need for hemodynamic support and longer time in the intensive care unit. However, in a study by Chen et al.^[20]^, the preoperative serum BNP concentration has not presented a significant association with the length of hospital stay and rehospitalization after elective CABG.

Regarding all-cause surgical mortality, our group found significant preliminary results in a group of patients underwent cardiac valve or CABG, demonstrating a significant association between higher preoperative serum BNP concentration and short-term mortality^[16,17]^. According to these findings, a systematic review by Litton & Ho^[21]^ showed a correlation between the preoperative serum BNP concentration and mortality, however due to the slightly relevant accuracy, the authors recommended in their study the clinical use of Judiciously.

Few studies specifically evaluated the preoperative BNP concentration in relation to mortality after OPCABG. In a study by Fox et al.^[22]^, the peak perioperative serum BNP concentration (preoperative up to the fifth postoperative day) was independently correlated with the combined outcome of hospitalization or death from heart failure in 5 years of follow-up. This study warned the need for clinical studies to evaluate whether a medical therapy focused on reducing the perioperative concentration of BNP would be able to reduce events after OPCABG.

At the present time, our study is original when it was designed methodologically with the specific purpose of evaluating the preoperative serum concentration of BNP and the short-term mortality after OPCABG, adding information to the scarce literature on the subject. An important prognostic function of this hormone in myocardial revascularization surgeries was shown. From the pathophysiological point of view, it is implicit that higher levels of BNP correlate with greater ventricular parietal tension, myocardial ischemia and its secretion and mechanism of action have a cardioprotective effect in the attempt to promote systemic and coronary arterial vasodilation, as well as venodilation with reduction of pre and post loading and improvement of cardiac index^[9,23]^.

The relevance of the topic is evident, since the natriuretic system is currently the focus of great therapeutic perspective in cardiovascular medicine. Although metanalysis still shows uncertainties regarding the use of BNP to guide treatment in patients with HF, the emergence of drugs (LCZ 696) acting directly on the enzyme system (neprilysin inhibitors) responsible for the degradation of BNP demonstrated a significant reduction in cardiovascular mortality in patients with heart failure and its clinical use is already authorized by regulatory agencies in some countries^[24,25]^.

Our group believes that the high preoperative serum BNP concentration characterizes a group of patients with a higher risk of death in myocardial revascularization surgeries and that new therapies guided by the level of BNP and drugs acting on its metabolic pathway may come to collaborate for a possible additional reduction of mortality after this surgical procedure.

A limitation of our study consists of a relatively small number of patients followed up, however, it can still be considered one of the largest studies among those related on the subject in the literature.

## CONCLUSION

The preoperative serum concentration of BNP is a strong independent predictor of all-cause mortality during the 30-day postoperative period in patients undergoing OPCABG.

**Table:** 

Authors' roles & responsibilities	
JAMJ	Substantial contributions to the conception or design of the work; or the acquisition, analysis, or interpretation of data for the work; drafting the work or revising it critically for important intellectual content; agreement to be accountable for all aspects of the work in ensuring that questions related to the accuracy or integrity of any part of the work are appropriately investigated and resolved; final approval of the version to be published;
MNM	Agreement to be accountable for all aspects of the work in ensuring that questions related to the accuracy or integrity of any part of the work are appropriately investigated and resolved; final approval of the version to be published;
MPF	Agreement to be accountable for all aspects of the work in ensuring that questions related to the accuracy or integrity of any part of the work are appropriately investigated and resolved; final approval of the version to be published;
MJFS	Agreement to be accountable for all aspects of the work in ensuring that questions related to the accuracy or integrity of any part of the work are appropriately investigated and resolved; final approval of the version to be published;
IHG	Agreement to be accountable for all aspects of the work in ensuring that questions related to the accuracy or integrity of any part of the work are appropriately investigated and resolved; final approval of the version to be published;
CCS	Agreement to be accountable for all aspects of the work in ensuring that questions related to the accuracy or integrity of any part of the work are appropriately investigated and resolved; final approval of the version to be published;
MFG	Agreement to be accountable for all aspects of the work in ensuring that questions related to the accuracy or integrity of any part of the work are appropriately investigated and resolved; final approval of the version to be published.
